# MicroRNA Profiling in Subventricular Zone after Stroke: MiR-124a Regulates Proliferation of Neural Progenitor Cells through Notch Signaling Pathway

**DOI:** 10.1371/journal.pone.0023461

**Published:** 2011-08-26

**Authors:** Xian Shuang Liu, Michael Chopp, Rui Lan Zhang, Tang Tao, Xin Li Wang, Haifa Kassis, Ann Hozeska-Solgot, Li Zhang, Charles Chen, Zheng Gang Zhang

**Affiliations:** 1 Department of Neurology, Henry Ford Hospital, Detroit, Michigan, United States of America; 2 Department of Physics, Oakland University, Rochester, Michigan, United States of America; Hôpital Robert Debré, France

## Abstract

**Background:**

The Notch signaling pathway regulates adult neurogenesis under physiological and pathophysiological conditions. MicroRNAs are small non-coding RNA molecules that regulate gene expression. The present study investigated the effect of miR-124a on the Notch signaling pathway in stroke-induced neurogenesis.

**Methodology and Principal Findings:**

We found that adult rats subjected to focal cerebral ischemia exhibited substantial reduction of miR-124a expression, a neuron specific miRNA, in the neural progenitor cells of the subventricular zone (SVZ) of the lateral ventricle, which was inversely associated with activation of Notch signals. In vitro, transfection of neural progenitor cells harvested from the SVZ of adult rat with miR-124a repressed Jagged-1 (JAG1), a ligand of Notch, in a luciferase construct containing the JAG1 target site. Introduction of miR-124a in neural progenitor cells significantly reduced JAG1 transcript and protein levels, leading to inactivation of Notch signals. Transfection of neural progenitor cells with miR-124a significantly reduced progenitor cell proliferation and promoted neuronal differentiation measured by an increase in the number of Doublecortin positive cells, a marker of neuroblasts. Furthermore, introduction of miR-124a significantly increased p27Kip1 mRNA and protein levels, a downstream target gene of the Notch signaling pathway.

**Conclusions:**

Collectively, our study demonstrated that in vivo, stroke alters miRNA expression in SVZ neural progenitor cells and that in vitro, miR-124a mediates stroke-induced neurogenesis by targeting the JAG-Notch signaling pathway.

## Introduction

The Notch pathway is a highly conserved regulatory signaling network [1] and has been linked to a variety of pathogenic conditions in human [2]. The Notch signaling pathway critically controls stem cell maintenance and cell fate determination [1], [3]. We and others have demonstrated that focal cerebral ischemia activates the Notch signaling pathway in neural progenitor cells localized to the subventricular zone (SVZ) of the lateral ventricle, leading to expansion of neural progenitor cells [3], [4], [5], [6].

MicroRNAs (miRNAs) are small, single-stranded RNA molecules of 21–23 nucleotides in length. miRNAs are encoded by genes from whose DNA they are transcribed, but miRNAs are not translated into protein; instead, each primary transcript (a pri-miRNA) is processed into a short stem-loop structure called a pre-miRNA and finally into a functional miRNA. Mature miRNA molecules are either fully or partially complementary to one or more messenger RNA (mRNA) molecules, and their main function is to down-regulate gene expression [7]. miRNAs have been recently shown to be crucial in regulating a variety of patho-physiological processes, including immune function, tumorigenesis, metabolism, and cell proliferation [8], [9], [10].

A relatively large number of these miRNAs are enriched in the brain [11]. Biological functions of brain miRNAs are emerging. miRNAs regulate neuronal and glial development and differentiation [12], [13]. MiR-124, a preferentially expressed miRNA in neurons, has recently been implicated in the positive modulation of the transitory progression of adult SVZ neurogenesis by repressing Sox9 [14], indicating that this specific miRNA is critical for the homeostasis of differentiation versus proliferation of adult neural progenitor cells [14], [15].

Studies in cancer cells show that several miRNAs cross-talk with the Notch pathway [16], [17], [18], [19], [20]. However, the role of miRNAs in the Notch pathway after stroke remains unclear. Understanding the interaction between miRNAs and the Notch signaling pathway in adult neural progenitor cells after stroke could potentially provide new therapies to enhance stroke-induced neurogenesis. Accordingly, the present study investigated miRNAs in mediating the Notch signaling pathway in neural progenitor cells after stroke.

## Results

### Stroke alters miRNA expression in SVZ neural progenitor cells

**Figure 1:**
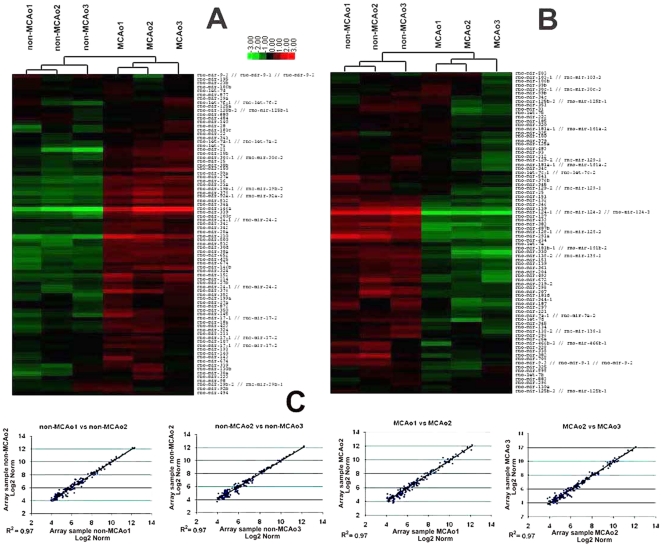
MicroRNA expression in SVZ neural progenitor cells. Hierarchical clustering of differentially expressed miRNAs (A, B). The data were from 6 individual microarrays (3 arrays per group). The individual expression signal of each miRNA in each array was clustered. The dendrograms (tree diagrams) show the grouping of miRNAs according to the order in which they were joined during the clustering. The color code in the heat maps is linear with green as the lowest and red as the highest. The miRNAs with increased expression are shown in red (A), whereas the miRNAs with decreased expression are shown in green (B). Correlation of the hybridization signal intensities of all the expressed miRNAs among three non-MCAo samples and MCAo showed few differences(C).

**Figure 2:**
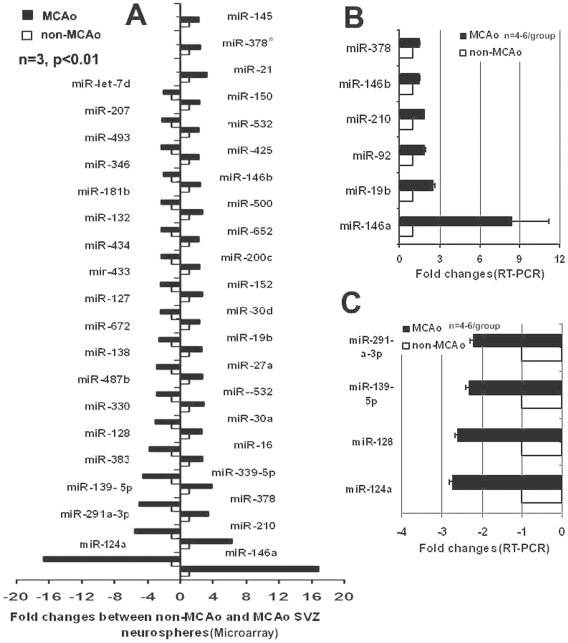
Validation of top differential expressions of miRNAs. Microarray data (A) show 18 and 21 of miRNAs with 2 fold changes (p<0.01) were found to be poorly and highly expressed, respectively, in ischemic neural progenitor cells (MCAo). N = 3 individual cultured SVZ cells/group. Real-time RT-PCR analysis shows ten of increased (B) and decreased (C) miRNAs detected on the microarray. Data are mean ± SE. N = 4–6 individual cultured SVZ cells/group.

To examine the expression profile of miRNAs after focal cerebral ischemia, we analyzed the global expression of mature miRNAs in cultured neural progenitor cells isolated from the SVZ in rats 7 days after right middle cerebral artery occlusion (MCAo, n = 3 individual cultured SVZ cells, Table S1). SVZ neural progenitor cells isolated from non-ischemic rats were used as a control group (n = 3). miRNA microarray platform was used to screen the expression profiles of miRNAs (Fig. 1A–1C, for more detailed, please see Figure S1). We found that 38 and 48 miRNAs in ischemic neural progenitor cells were at least 1.5 fold upregulated and 1.5 fold downregulated, respectively (P<0.05, Table S1). Among them, 18 of these were found to be poorly expressed, whereas 21 of these were highly abundant in the ischemic neural progenitor cells with 2 fold or greater changes (P<0.01, Fig. 2A).

To analyze the likely role of these miRNAs in neural progenitor cells, a biological function analysis was performed on the miRNAs in the SVZ cells, which were de-regulated more than two fold with a p<0.01 (Fig. 2A). Twenty-one upregulated miRNAs and eighteen downregulated miRNAs were chosen for further pathway analysis using DIANA – mirPath software (http://diana.cslab.ece.ntua.gr/pathways/) [21]. The top 10 ranked biologic functions associated with commonly upregulated miRNAs include regulation of axon guidance, the MAPK signaling pathway, focal adhesion, ErbB signaling pathway, actin cytoskeleton, Wnt signaling pathway, GnRH signaling pathway, insulin signaling pathway, glioma, and renal cell carcinoma (Table S2). The top 10 ranked biologic functions associated with commonly downregulated miRNAs included axon guidance, the MAPK signaling pathway, pancreatic cancer, focal adhesion, renal cell carcinoma, TGF-beta signaling pathway, insulin signaling pathway, Wnt signaling pathway, mTOR signaling pathway, prostate cancer, adhere junction, the ErbB signaling pathway, glioma, and regulation of actin cytoskeleton (Table S2).

### Validation of miRNA expression in SVZ neural progenitor cells after MCAo

**Figure 3:**
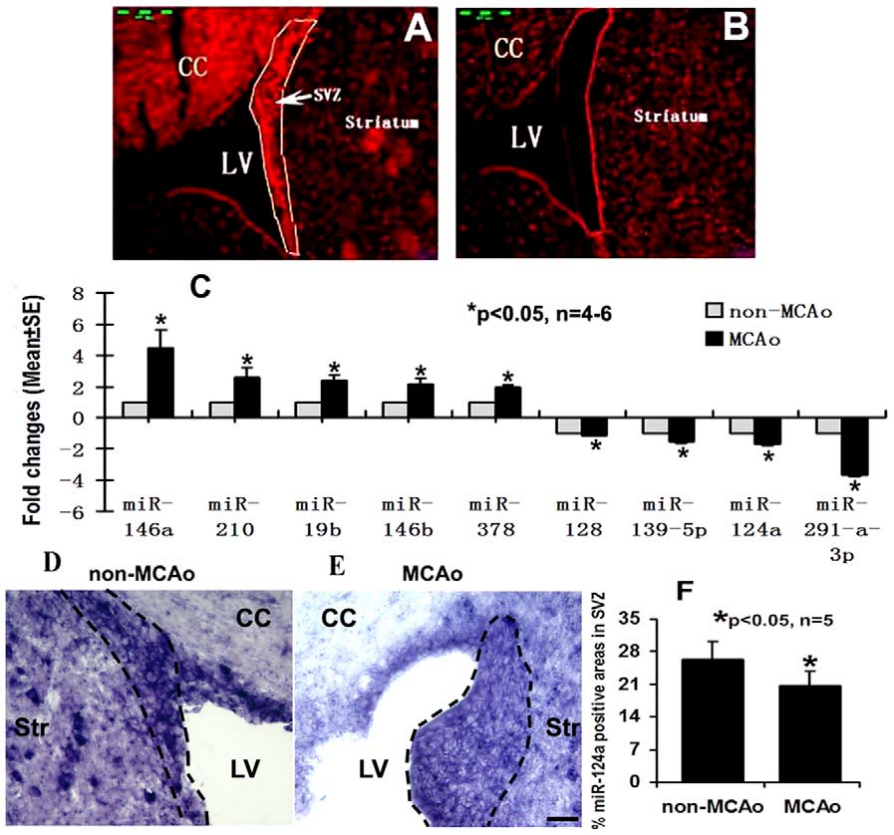
Expression of miRNAs in vivo. Panels A and B show SVZ cells before (A) and after (B) laser capture microdissection (LCM). Panel C shows real-time RT-PCR results of miRNA expressions in non-ischemic (non-MCAo) and ischemic (MCAo) SVZ cells isolated by LCM. N = 4–6 rats/group. In situ hybridization with digoxigenin (DIG)-labeled RNA probes shows miR-124a signals in non-ischemic (D, outlines) and ischemic (E, outlines) SVZ. Panel F shows quantitative data of miR-124a. CC = corpus callosum; LV = lateral ventricle; Str = striatum; SVZ = subventricular zone. N = 5 rats/group. Scale bar = 40 µm.

Using Taqman probes and quantitative real-time RT-PCR (qPCR), which detect mature miRNAs, we verified the most altered miRNAs detected on the microarray in neural progenitor cells after MCAo (Fig. 2B and 2C). Among them, miR-124a was significantly decreased in ischemic SVZ neural progenitor cells. Since there may be biological differences between cells obtained in vivo and from cultures, we analyzed miRNA profiles in SVZ neural progenitor cells isolated from the brain tissue by laser capture microdissection (LCM, Fig. 3A and B) and found a significant reduction of miR-124a in these cells 7 days after stroke (Fig. 3C). In addition, the neural progenitor cells isolated by LCM exhibited increases in miR-146a, miR-146b, miR-210, miR-19b and miR-378 and decreases in miR-128, miR-291a-3p, and miR-139-5p (Fig. 3A to 3C), which are consistent with the array data findings. Although there is magnitude discrepancy of gene expression between the array and PCR data for miRNAs listed above, both methods demonstrated that stroke significantly change miRNA expression. In addition to differences between SVZ cells isolated from ex vivo and cultured SVZ cells, one of the reasons for the discrepancy may lie in the different platforms employed to detect different miRNA amplicons [22].

### MiR-124a in SVZ progenitor cells mediates stroke-induced neurogenesis

In situ hybridization with digoxigenin (DIG)-labeled LNA probes that target the mature form of miR-124a shows the presence of miR-124a signals in non-ischemic SVZ cells (Fig. 3D), which is consistent with a published study [14]. However, 7 day ischemia substantially reduced miR-124a in SVZ cells (Fig. 3E, F) compared to miR-124a signals in the contralateral SVZ (Fig. 3D, F), which is concomitant with substantial increases in neural progenitor cell proliferation 7 days after stroke, as previously demonstrated [5], [23]. These data suggest that miR-124a could regulate progenitor cell proliferation after stroke. We therefore, examined the effect of delivery of miR-124a on neural progenitor cell proliferation.

**Figure 4:**
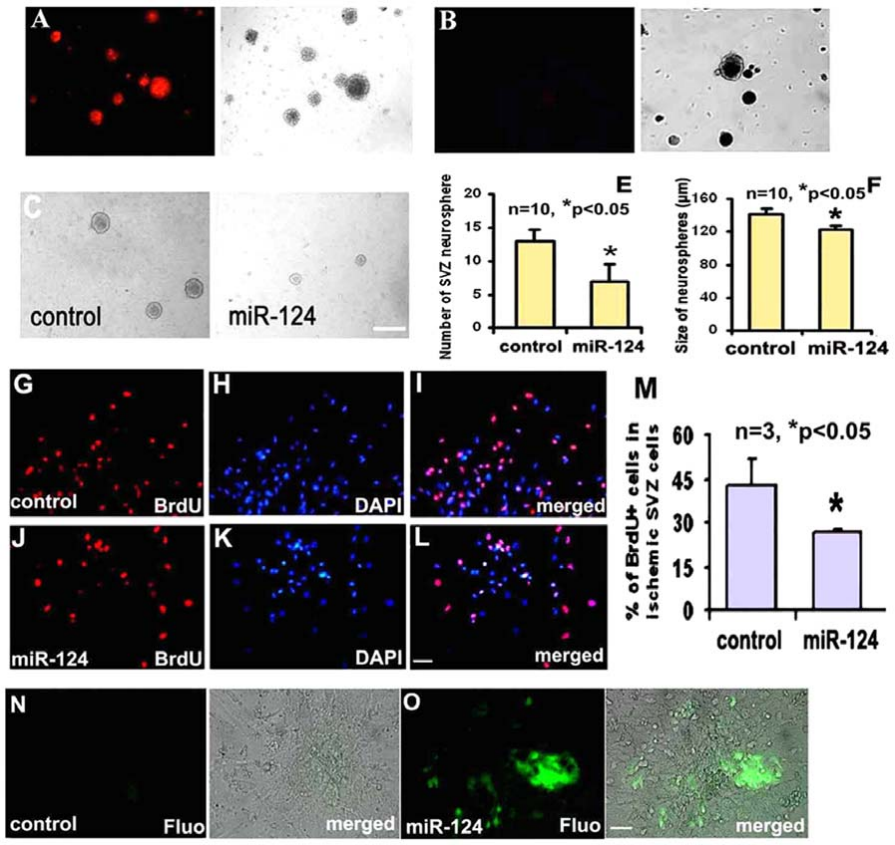
The effect of miR-124a on proliferation and differentiation of neural progenitor cells. Microscopic images acquired under fluorescent and bright fields after introduction of miRNA mimic indicator conjugated with Dy547 show more than 90% SVZ neural progenitor cells exhibiting red fluorescence, indicating robust delivery efficiency (A). No red fluorescence was observed in SVZ neural progenitor cells in the presence of miRNA mimic indicator but without nanoparticles (B). Panels C and D show neurospheres cultured in the proliferation medium after introduction of miR mimic controls (C) and miR-124a mimics (D), while panels E and F show quantitative data of number (E) and size (F) of neurospheres delivered with mimic controls (control) or miR-124a mimics (miR-124a) in the proliferation medium. Panels G to M show BrdU immunoreactive cells after transfection with miRNA mimic controls (G to I, and M) and miR-124a mimics (J to L, and M). Panels N and O show DCX-EGFP SVZ cells cultured in the differentiation medium after introduction of miRNA mimic controls (N) and miR-124a mimics (O). Scale bar = 100 µm in D and 20 µm in L and O.

To deliver miRNA into neural progenitor cells, a newly developed nanoparticle-mediated method was employed [24], To verify the delivery efficiency of nanoparticles, miR mimic indicator (cel-miR-67) which was conjugated with Dye548 was introduced into SVZ neural progenitor cells and approximately 90% progenitor cells were observed to be red fluorescence 10 h after delivery (Fig. 4A). However, no cell exhibited red fluorescence in the absence of nanoparticles, suggesting the specific and efficient delivery of miRNA into progenitor cells by nanoparticles (Fig. 4B). In addition, introduction of nanoparticles to SVZ cells did not cause an increase in TUNEL positive cells compared with SVZ cells without introduction of nanoparticles (data not shown). We then delivered nanoparticles with miR-124a mimics into ischemic SVZ neural progenitor cells. Using a neurosphere assay in which single ischemic SVZ cells (10 cells/µl) were incubated in the growth medium, we examined the effect of miR-124a on cell proliferation. Introduction of miR-124a mimics in ischemic neural progenitor cells significantly (P<0.05) decreased the numbers and size of neurospheres (Fig. 4C–4F) and the number of BrdU-positive cells (Fig. 4G–M) compared with cells delivered with miRNA mimic controls. Together, these results showed that nanoparticle-delivered miR-124a suppressed ischemia-induced progenitor cell proliferation.

To examine the effect of miR-124a on progenitor cell differentiation, SVZ cells after introduction of miR-124a mimics or mimic controls were cultured under the differentiation media. Real-time RT-PCR analysis revealed that introduction of miR-124a strikingly increased the expression of DCX (4.5±0.3 vs 1.0±0.2 in the control, n = 3, p<0.05), a marker of migrating neuroblasts, but did not significantly affect GFAP mRNA levels compared with the cells transfected with mimic controls (1.3±0.2 vs 1.0±0.2 in the control, n = 3, p>0.05). Consistent with mRNA results, introduction of miR-124a mimics into SVZ neural progenitor cells isolated from the DCX-eGFP transgenic mouse resulted in two fold increases in DCX-eGFP neurospheres in the differentiation medium (9.5±3.6% in miR-124a group vs 5.1±2.9% mimic control group, n = 12, Fig. 4N and O, p<0.05). These data suggest that increases of miR-124a promote neuronal differentiation.

### MiR-124a regulates Notch signaling pathway

**Figure 5:**
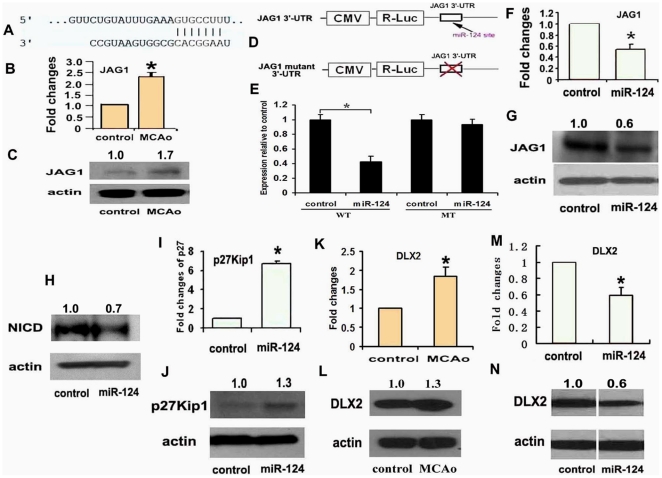
miR-124a targets the 3′-UTR of JAG1. Panel A shows sequence of a potential miR-124a binding site in the rat. Real-time RT-PCR (B) and Western blot (C) show mRNA and protein levels, respectively, of JAG1 in non-ischemic (control) and ischemic (MCAo) SVZ neural progenitor cells. Schematic representation (D) of a miR-124a reporter vector containing a CMV promoter driving the expression of luciferase cDNA fused to the JAG1 3′-UTR (pMIR-JAG1-3′-UTR) or to a mutated JAG1 3′-UTR (pMIR-JAG1-mu-3′-UTR). Panel E shows relative luciferase activity of constructs containing the pMIR-JAG1-3′-UTR or pMIR-JAG1-mu-3′-UTR introduced into NIH 3T3 cells in the presence of miR-124a mimics and mimic controls. A pRL-TK vector was transfected into the cells along with the pMIR-JAG1-3′-UTR or pMIR-JAG1-mu-3′-UTR used as an internal control. Panels F to L show real-time RT-PCR data of JAG1 (F) and p27Kip1 (I) mRNA levels and immunoblotting of JAG1 (G), NICD (H), and p27Kip1 (J) protein levels in ischemic neural progenitor cells delivered with mimic controls (control) or miR-124a mimics (miR-124a). Panels K and L show mRNA and protein levels, respectively, of DLX2 in non-ischemic (control) and ischemic (MCAo) neural progenitor cells. Panels M and N show that nanoparticle-delivered miR-124a mimics into ischemic neural progenitor cells (miR-124a) suppressed DLX2 mRNA (M) and protein (N) levels compared to the cells delivered with control mimics (control). N = 3 individual cultured SVZ cells/group, * p<0.05.

Previous studies have shown that under non-ischemic conditions, miR-124 targets Sox9, JAG1 and DLX2, and that miR-124 mediates neurogenesis by repressing Sox9 in SVZ cells [14], [25]. However, stroke did not significantly increase Sox9 levels in SVZ neural progenitor cells (1.2±0.2 in ischemic vs 1.0±0.1 in non-ischemic, n = 3, p = 0.23). JAG1 is a ligand of the transmembrane protein Notch receptors [26], and the Notch signaling pathway mediates stroke-induced neurogenesis [1], [4], [5], [6]. The role of miR-124a in mediating the Notch signaling pathway has not been investigated in ischemic neural progenitor cells. Our real-time RT-PCR and Western blot analysis showed that stroke increased JAG1 mRNA and protein levels in SVZ neural progenitor cells, which was negatively correlated with miR-124a signals (Fig. 5A, B, C and Fig. 2). To further validate this computational finding that miR-124a may negatively regulate JAG1, we generated a luciferase construct harboring the 3′-UTR fragment of JAG1 containing a broadly conserved binding site of miR-124a (Luc-JAG1, Fig. 5D) and a mutant luciferase construct with deletion of the binding site (Luc-JAG1-mu, Fig. 5D). Luciferase assay showed that miR-124a significantly repressed the luciferase activity in the 3T3 cell line transiently transfected with Luc-JAG1, compared with cells transfected with Luc-JAG1-mu (Fig. 5E), which is consistent with previous findings that JAG1 is a putative target of miR-124a [14].

We then examined the effect of miR-124a on JAG1 expression in ischemic SVZ neural progenitor cells. Ischemic progenitor cells were delivered with miR-124a mimics and incubated in the growth medium for 3 days. JAG1 and Notch intracellular domain (NICD) were assayed by real-time RT-PCR and Western blot. Compared with the miRNA mimic control, nanoparticle-delivered mature miR-124a resulted in a substantial decrease of JAG1 transcript and protein (Fig. 5F and G). Introduction of miR-124a also substantially decreased NICD levels (Fig. 5H) compared with the mimic control group. Furthermore, introduction of miR-124a mimics increased p27Kip1 transcripts approximately 6.7 fold (Fig. 5I) and protein expression about 1.3 fold (Fig. 5J), compared with the miRNA mimic control group, which is consistent with previous findings that p27Kip1 is a negative target gene of Notch signaling pathway [27], [28]. Collectively, these data indicate that miR-124a targets the JAG1-Notch signaling pathway in ischemic neural progenitor cells.

In addition, real-time RT-PCR and Western blot analysis showed that DLX2, a homeobox gene [29], mRNA and protein levels, respectively, were greatly increased in ischemic SVZ neural progenitor cells, which were concurrent with downregulation of miR-124a level (Fig. 5K and L). Introduction of miR-124a mimics into ischemic neural progenitor cells significantly decreased levels of mRNA and DLX2 protein (Fig. 5M and N).

## Discussion

We demonstrated that stroke altered expression profiles of multiple miRNAs in SVZ neural progenitor cells and that introduction of miR-124a inhibited ischemic neural progenitor cell proliferation and promoted the neuronal differentiation of the progenitor cells by targeting JAG1. These data provide new insights into the molecular mechanisms underlying stroke-induced neurogenesis.

MicroRNAs regulate biological function of neural stem cells in developing and adult CNS [11], [12], [13], [14]. However, the role of miRNAs in mediating stroke-induced neurogenesis has not been investigated. The present study showed that stroke substantially altered expression profiles of miRNAs in SVZ neural progenitor cells and that many of the altered miRNAs could potentially regulate the MAPK and Wnt signaling pathways known to regulate neurogenesis. These data suggest that miRNAs are actively involved in stroke-induced neurogenesis and our data also provide bases to further investigate biological function of the altered miRNAs in stroke-induced neurogenesis.

MiR-124 is a neuron-specific miRNA and regulates neurogenesis in SVZ neural progenitor cells of adult normal mice [14], [15], [30], [31]. Our in vivo and in vitro data showed that down-regulation of miR-124a by stroke enhances progenitor cell proliferation, while introduction of miR-124a attenuates stroke-increased proliferation and increases neuronal differentiation, indicating that miR-124a in SVZ neural progenitor cells could mediate stroke-induced neurogenesis. Under non-ischemic conditions, miR-124 promotes transition from the transit amplifying SVZ progenitor cells to the neuroblasts by physiologically targeting Sox9 [14], [25]. Sox9 is required for the generation and maintenance of adult SVZ neural stem cells [32]. However, the present study showed that stroke did not significantly change Sox9 expression. Instead, our data provide several lines of evidence to demonstrate that in vitro, miR-124a mediates stroke-induced neurogenesis by targeting the JAG1 in neural progenitor cells. Introduction of miR-124a mimics suppressed JAG1 expression, whereas mutation of the JAG1 binding site abolished the effect of miR-124a on JAG1 expression. There are two predicted targets in the full length 3′-UTR of JAG1 (www.targetscan.org). Published data show that miR-124a significantly decreased luciferase activity in 3′-UTR of JAG1, which includes two target sites [14]. Thus, the effect of miR-124a on JAG1 is specific. In addition, introduction of miR-124a attenuated activation of NICD and upregulated p27Kip1, a target gene of Notch signaling pathway, indicating that miR-124a mediates activation of the Notch pathway. The Notch receptors are transmembrane proteins activated by Delta and JAG ligands [26]. The Notch signaling pathway is essential to maintain the SVZ neural stem cell niche of adult rodent [33]. We and others previously demonstrated that the Notch signaling pathway mediates stroke-induced neurogenesis [3], [5], [6]. Activation of Notch signals by stroke increases proliferation of SVZ neural progenitor cells, while down-regulation of the Notch signals promotes neural progenitor cell differentiation into neuroblasts [5], [6]. Administration of a Notch ligand, Dll4, increases neurogenesis in the ischemic brain [3]. We now demonstrate that miR-124a regulates activation of the Notch signaling pathway in SVZ neural progenitor cells. However, in vivo experiments are warranted to demonstrate the cause effect of miR-124a through targeting JAG1 on stroke-induced neurogenesis, although our in vivo data showed that down-regulation of miR-124a by stroke was inversely correlated to upregulation of JAG1. In addition to miR-124a, stroke downregulates miR-9 and miR-139 in neural progenitor cells and these miRNAs have been predicted to target Notch and Hes1 [34] (www.targetscan.org), suggesting that miR-9 and miR-139 could also regulate the Notch signaling pathway after stroke. In cancer cells, miR-326 [17], miR-34a [18], miR-206 [19], let-7 [35] have been shown to regulate the Notch signaling pathway. Therefore, we expect that there are multiple miRNAs acting in concert with miR-124a to fine-tune the Notch pathway in stroke-induced neurogenesis.

The homeobox transcription factor DLX2 regulates generation of interneurons in the embryo [36] and promotes neurogenesis in the postnatal SVZ [37]. The present study showed that stroke upregulated DLX2, which was concurrently with reduction of miR-124a, whereas miR-124a mimics reduced DLX2 expression in ischemic neural progenitor cells. MiR-124 targets DLX2 [14]. Thus, downregulation of miR-124a by stroke could upregulate DLX2 expression, leading to proliferation of SVZ neural progenitor cells. In general, miRNAs can regulate numerous target mRNAs at the post-transcriptional level [38], [39]. Data are emerging that some individual miRNAs also regulate target mRNAs at transcriptional levels [40], [41]. Whether miR-124 regulates JAG1 at the transcriptional level is warranted for future studies.

In addition to its role in neurogenesis, upregulation of miR-124 suppresses development of autoimmune encephalomyelitis by inactivation of macrophages [42]. Inflammation contributes to ischemic cell damage [43]. Ischemia substantially changes miRNA expression profiles during the acute stage [44], [45], [46], [47]. Inhibition of miR-145 and upregulation of miR-21 have neuroprotective effects [46], [48]. Thus, investigation of the effects of miR-124 on inflammation in the ischemic brain is warranted.

In summary, our study revealed the cell–specific pattern of miRNAs in SVZ neural progenitor cells after stroke. Downregulation of miR-124a induces JAG1 expression in the SVZ neural progenitor cells after stroke and thereby promotes neural progenitor cell proliferation. As neurogenesis is related to the behavioral recovery of stroke [49], miR-124a could potentially be used as a therapeutic target to amplify endogenous neurogenesis after stroke.

## Materials and Methods

All experimental procedures were carried out in accordance with the NIH Guide for the Care and Use of Laboratory Animals and approved by the Institutional Animal Care and Use Committee of Henry Ford Hospital (IACUC approval number: 1069).

### Animal model of middle cerebral artery occlusion (MCAo)

Male Wistar rats (3–4 months) were employed in this study. The right middle cerebral artery (MCA) was occluded by placement of an embolus at the origin of the right MCA, as previously described [50]. In this model, MCA occlusion (MCAo) evokes a peak increase of neurogenesis 7 days after stroke [51]. Therefore, all rats were sacrificed 7 days after MCAo.

A doublecortin-enhanced green fluorescent protein (DCX-eGFP) mouse line was purchased from the Mutant Mouse Regional Resource Center. We have verified specificity of DCX-eGFP expressing cells in the adult DCX–eGFP SVZ [23], [52].

### SVZ cell culture

SVZ neural progenitor cells were isolated from adult rats and DCX–EGFP mice, as previously described [5], [53]. The cells were plated at a density of 2×10^4^ cells/µl in the growth medium, which contains DMEM/F-12 medium (Invitrogen Corporation, Carlsbad, CA, USA), 20 ng/ml epidermal growth factor (R&D Systems, Minneapolis, MN, USA), and basic fibroblast growth factor (R&D Systems). DMEM/F-12 medium contains L-glutamine (2 mmol/liter), glucose (0.6%), putrescine (9.6 mg/ml), insulin (0.025 mg/ml), progesterone (6.3 ng/ml), apotransferrin (0.1 mg/ml), and sodium selenite (5.2 ng/ml). The generated SVZ neurospheres (primary spheres) were passaged by mechanical dissociation and reseeded as single cells at a density of 20 cells/µl. Passaged 1 SVZ cells were employed for assay of the miRNA array. Other experiments used cultured SVZ neural progenitor cells which were passaged less than 5 to avoid the likely genetic variation of progeny [54].

### miRNA array assay and analysis

Cultured SVZ cells isolated from 3 rats were pooled for each miRNA array analysis and three individually cultured SVZ cells were collected for non-MCAo and MCAo groups. Total RNAs were extracted using mirVana™ miRNA Isolation Kit (Applied Biosystems, Foster City, CA, USA) in accordance with the manufacturer's procedure. RNA samples were analyzed by Biomarker Incoporation (Precision Biomarker Resources Inc, Evanston, IL, USA) using GeneChip® miRNA Array (Affymetrix Inc, Santa Clara, CA, USA) containing 46,228 probes comprising 7,815 probe sets, including controls. Among them, 352 probe sets are specific for rat miRNAs. Content is derived from the Sanger miRBase miRNA database v11.

For microarray data analysis, quality control of the total RNA samples was assessed using UV spectrophotometry and agarose gel electrophoresis. The samples were DNAse digested and low-molecular weight (LMW) RNA was isolated by ultrafiltration through YM-100 columns (Millipore, Billerica, MA, USA) and subsequent purification using the RNeasy MinElute Clean-Up Kit (Qiagen, Valencia, CA, USA). The LMW RNA samples were 3′-end labeled with biotin dye using the FlashTag™ Biotin RNA Labeling Kits (Genisphere, Hatfield, PA, USA). Labeled LMW RNA samples were hybridized to the MicroRNA microarrays according to conditions recommended in the Flash Taq RNA labeling Kit manual. The microarrays were scanned on an Axon Genepix 4000B scanner (Affymetrix Inc), and data were extracted from images using GenePix V4.1 software. miRNA QC Tool (Affymetrix Inc), a software for data summarization, Log2 transformation, normalization and quality control, was used. Microarray data is MIAME compliant and the raw data has been deposited in the Gene Expression Omnibus (GEO) database.

### Bioinformatics analysis

To assay the gene targets of differentially expressed miRNAs, we used three of the leading miRNA target prediction algorithms miRanda (http://microrna.sanger.ac.uk/sequences/), PicTar (http://pictar.mdc-berlin.de/), TargetScan (http://www.targetscan.org/). To perform an enrichment analysis of predicted target genes of miRNAs in biological pathways, DIANA-mirPath, a web-based application [55], was used. This software analyses lists genes in the context of known biological response and regulatory networks as well as other higher-order response pathways.

### In Situ Hybridization

In situ hybridization was performed according to a published protocol [56]. Briefly, rats subjected to 7 day MCAo or sham surgery were sacrificed under anesthesia by intracardial TBS-paraformaldehyde perfusion. Coronal brain sections (20 µm thick) from each rat were post-fixed and acetylated by incubating in acetic anhydride/triethnolamine solution followed by washes in 1× PBS. The sections were incubated in hybridization solution (50% formamide, 5× SSC, 200 µg/mL yeast tRNA, 500 µg/mL salmon sperm DNA, 0.4 g Roche blocking reagent, and 5× Denhardt's solution) at room temperature for 2 h. The sections were incubated overnight in hybridization solution containing 3 pmol of digoxin (DIG)-labeled LNA MiRCURY probes (Exiqon Inc, Woburn, MA, USA) at below −20° predicted Tm value of the probe used. The sections were washed at 55°C for 30 m in 1× SSC and for 10 min in 0.1 M Tris-HCl buffer (pH 7.5) and incubated in the blocking solution (10% fetal calf serum in 0.1 M Tris-HCl buffer) for 1 h at room temperature followed by labeling with anti-DIG-FAB peroxidase (POD, Roche Applied Science, Indianapolis, IN, USA) for 1 h at room temperature. The signals were amplified using the Individual Indirect Tyramide Reagent Kit (PerkinElmer Life Science, Waltham, Massachusetts, USA), according to the protocol [56]. Alkaline phosphatase was used for the detection of the miRNA signals.

For semiquantitative measurements of miR-124a signals, one coronal sections/rat (N = 5 rats) were employed. The SVZ area was digitized with a 20× objective (BX20 Olympus Optical) using a 3-CCD color video camera (DXC-970 MD; Sony, Tokyo, Japan) interfaced with a MCID image analysis system. The entire SVZ area and areas with miR-124a signals in the SVZ were measured, as described previously [57]. Data are presented as a percentage of miR-124a signals within the SVZ.

### Laser Capture Microdissection (LCM)

LCM was performed according to our published protocol [53], [58]. Briefly, frozen brain coronal sections (8 µm) stored at −80°C were immediately immersed in acetone for 2 min of fixation and air-dried for 30 s. After a brief rinse with 0.1% diethylpyrocarbonate-treated phosphate buffered saline, sections were stained with propidium iodide dye (1∶5000 dilution, Sigma Aldrich, Louis, MO, USA) for 2 min and rinsed with phosphate buffered saline twice. Sections were then air-dried under laminar flow for 10 min and immediately used for LCM. Dense SVZ cells on sections stained by propidium iodide were readily distinct from the ependymal cells that have cilia along the lateral wall of the lateral ventricle and from the adjacent striatal cells. In the non-ischemic rat, the dorsal and ventral SVZ of the lateral wall was defined as a 20–30 µm-wide zone approximately of 2–3 cell bodies immediately adjacent to ependymal cells, whereas in the ischemic rat, the SVZ was expanded to a 60–80 µm-wide zone. Propidium iodide-positive cells within the SVZ were dissected with a Leica AS LMD System (Leica Microsystems Inc). The excised cells fell into a collection tube under gravity, ensuring contamination-free processing and minimizing sample damage. Eppendorf tubes containing 40 µl of lysis buffer were stored at −80°C before miRNA isolation. Approximately 1,000 cells were isolated in the SVZ from each animal.

### Quantification of mRNA by real-time qRT-PCR

RNAs extracted from the SVZ were reverse transcribed using M-MLV reverse transcriptase (Invitrogen). 2 µg of RNA from each sample was reverse transcribed at 42°C for 30 min with 1 µg of Oligo^dT^ or specific primers, 5× first strand buffer, 100 mM DTT, 10 mM dNTP, RNAsin (Invitrogen) and M-MLV. cDNAs were checked for their optimum dilution in subsequent real-time qRT-PCR reactions. PCR reaction mixtures included cDNAs in optimum dilution, the SYBR Green qPCR Master mixture (Applied Biosystem), 10 µM primers, in a total reaction volume of 20 µl. Expression profiling was done with dissociation curves using ABI 7000 (Applied Biosystem). Cycling parameters were 95°C for 4 min followed by 40 cycles of 20°C/s temperature transition rate up to 95°C (30 s), 62°C (45 s), followed by melting curve analysis. All reactions were performed in triplicate with reference dye normalization (β-actin) and the median Ct (Cycle threshold) value was used for analysis. Please see Table S3 for detailed primer sequences. The relative abundance of each target was calculated using the 2−^ΔΔCt^ method [59].

### Quantification of mature miRNAs by real-time qRT-PCR

Individual reverse transcription and TaqMan® microRNA assays were performed on an Applied Biosystems 7000 Instrument (Applied Biosystem). 15 µL Reverse transcription reactions consisted of 1–10 ng Total RNA isolated with TRIzol (Qiagen), 5 U MultiScribe Reverse Transcriptase, 0.5 mM each dNTPs, 1× Reverse Transcription buffer, 4 U RNase Inhibitor, and nuclease free water. Reverse transcription reactions were incubated at 16°C for 30 min, 42°C for 30 min, 85°C for 5 min, and then stored at 4°C until use in TaqMan assays. 20 µL TaqMan real-time PCR reactions consisted of 1× TaqMan Universal PCR Master Mix No AmpErase UNG, 1× TaqMan miRNA assay, 1.33 µL of undiluted cDNA, and nuclease free water. Each TaqMan assay was done in triplicate for each sample tested. Relative quantities were calculated using the 2−^ΔΔCt^ method with U6 snRNA TaqMan miRNA control assay (Applied Biosystem) as the endogenous control and calibrated to the wild type samples [59]. Three independent experiments were performed. Reactions were run with the Standard 7000 default cycling protocol without the 50°C incubation stage, with reactions incubated at 95°C 10 min, followed by 40 cycles of 95°C 15 sec, 60°C 1 min. Fluorescence readings were collected during the 60°C step.

### Nanoparticle-mediated miRNA Transfection

To efficiently introduce the miRNA into neural progenitor cells, N-TER Nanoparticle Transfection System was employed [24]. Briefly, N-TER Peptide was diluted into water in a sterile tube and incubated in a sonicating water bath at maximum output and continuous power for 3–5 minutes. Then 5 mM miR-124a mimic (mature sequence: UAAGGCACGCGGUGAAUGCC, Dharmacon Inc, Chicago, IL, USA) or miRNA mimic control (Dharmacon Inc) was diluted with N-TER Buffer in a sterile tube. The Nanoparticle Formation Solutions were prepared by combining the appropriate diluted miRNA solutions with diluted N-TER Peptide solutions, and incubated the tubes containing the Nanoparticle Formation Solutions (combined miRNA and N-TER Peptide solutions) at room temperature for 20 minutes to allow the nanoparticles to form. A solution of Nanoparticle Formation Solutions was mixed in 1400 µL of growth medium. This solution was added to the cells and slightly agitated to mix. After 24 h at 37°C, the solution was removed from the cells and replaced with 37°C growth medium or differentiation medium.

### Neurosphere Assay

A neurosphere assay was employed to investigate the effect of miR-124a on SVZ neural progenitor cells. The assay has been widely used by us and others as a valuable tool for investigating the biology of neural progenitor cells [53], [60], [61]. To examine the effects of miRNAs on the proliferation of SVZ neural progenitor cells, two methods were used [53], [61]. To analyze the formation of secondary neurospheres, SVZ cells were gently triturated with a fire-narrowed Pasteur pipette, spun down at 400 rpm for 3 min, and then seeded at a density of 10 cells/ml in 96 well plates. The number and size of neurospheres were measured at 7 days in vitro. To analyze cell proliferation, single cells at a density of 10 cells/µl were incubated in the growth medium for 3 days, and bromodeoxyuridine (BrdU, 30 µg/ml, Sigma Aldrich), the thymidine analog that is incorporated into the DNA of dividing cells during S-phase, was added 18 h before the termination of incubation. BrdU positive cells were measured (see blow for quantification).

To examine the effects of miRNAs on SVZ cell differentiation, neurospheres were plated directly onto laminin-coated glass coverslips in DMEM/F-12 medium containing 2% fetal bovine serum without bFGF and EGF, which is referred to as a differentiation medium, in the presence of miRNA mimic. Every 4 days, half of the medium was replaced with fresh medium. Incubation was terminated 10 days after plating. The cells were processed to mRNA analysis for identifying genotype of SVZ cells [53].

### Immunocytochemistry and Quantification

Immunofluorescent staining was performed on cultured cells. Mouse anti-BrdU (1∶100; Boehringer Mannheim, Indianapolis, IN, USA) was used as the primary antibody in the present study. Cultured cells were fixed in 4% paraformaldehyde for 20 min at room temperature. Nonspecific binding sites were blocked with phosphate-buffered saline with 1% bovine serum albumin goat serum for 1 h at room temperature. The cells were then incubated with the primary antibodies listed above and with CY_3_-conjugated secondary antibodies. Nuclei were counterstained with 4-,6-diamidino-2-phenylindole (1∶10,000, Vector Laboratories, Burlingame, CA, USA).

The number of BrdU-positive cells as well as total 4_,6-diamidino-2-phenylindole (DAPI) nuclei was counted under a 40× objective (IX71; Olympus Optical, Tokyo, Japan), and the percentage of BrdU/DAPI was determined. For all measurements, we counted at least 500 cells from three wells/group (n = 3 individual cultured cells).

### Luciferase activity assay

There are at least two predicated target sites for miR-124a in the entire 3′-UTR of Jagged-1 (JAG1) (www.targetscan.org). As we had difficulty to amplify the full 3′-UTR of JAG1, a 286 bp fragment of JAG1 3′-UTR from the rat was amplified by PCR using the primers 5′-CGACTAGTGGTTTTATGATGACGTA-3′ and 5′-CGAAGCTT GAATGATGTTTTAAGGC-3′. The fragment, which contains a broadly conserved motif in the vertebrates for miR-124a (www.targetscan.org) was cloned downstream of the luciferase gene in pMIR-REPORT luciferase vector (Ambion, Austin, TX, USA). This construct, named pMIR-JAG1, was used for transfection in 3T3 cell line (ATCC, Manassas, VA, USA). To generate a mutant containing a deletion of the miR-124a target sequence, PCR and appropriate primer sets (F: 5′-GACGTA CAAGTAGTTCTGTATTTGAAATGCAGCTCAGAACC and R: 5′-GGTTCTGAGCTGCA TTTCAAATACAGAACT ACTTGTACGTC) were used to amplify JAG1-mutant. PCR product was subcloned into the CMV promoter luciferase reporter pMIR-REPORT (Ambion) using SpeI and Hind III restriction enzymes (Invitrogen).

3T3 cells were cultured in 96 well plates and each transfected with 0.15 µg of either pMIR-JAG1 (pMIR-JAG1 mu) or pMIR-REPORT together with 0.05 µg of pRL-TK vector (Promega, Madison, WI, USA) containing Renilla luciferase and miR-124a or negative control miRNA mimics. Transfection was done using Lipofectamine 2000 and Opti-MEM I reduced serum medium (Invitrogen). Forty-eight hours after transfection, firefly and Renilla luciferase activity were measured using Dual luciferase assay kit (Promega) with plate reader (Perkin Elmer, Waltham, MA, USA). The results were expressed as relative activity. Each transfection was repeated twice in triplicate.

### SDS-PAGE and Western blot

Cells were lysed in RIPA buffer, and lysate was sonicated and then centrifuged for 10 min at 12,000 rpm to remove cell debris. Protein concentrations were determined using a BCA assay (Thermo Scientific, Waltham, MA, USA). Equal amounts of proteins were then separated by SDS-PAGE and transferred to a nitrocellulose membrane. Membrane was probed with an appropriate primary antibody and a secondary antibody conjugated to horseradish peroxidase. The following antibodies were utilized: β-actin (1∶10,000 dilution, Millipore), distalless (DLX)2 (1∶500 dilution, Chemicon), NICD (1∶500 dilution, Cell Signaling, Danvers, MA, USA), JAG1 (1∶500 dilution, Chemicon), p27Kip1 (1∶500 dilution, Santa Cruz). Proteins were visualized by enhanced chemiluminescence (Thermo Fisher Scientific, Rockford, IL, USA). Three independent experiments were performed and one representative result was shown.

#### Statistical Analysis

The data are presented as mean ± SE. Independent sample t-test was used for two-group comparisons from the non-MCAo and MCAo samples. One-way analysis of variance followed by Student-Newman-Keuls test was performed for multiple sample analysis. A value of p<0.05 was taken as significant.

## Supporting Information

Figure S1 Cluster diagram for detected microRNA from Affymetrix microRNA microarray experiment. MiRNA probe expression values (Log2 transformed & normalized microarray probe intensities) of detected miRNA in either MCAo non-MCAo samples were median centered. Each column represents a single sample, and each row represents a single miRNA probe. Green squares represent lower than median levels of gene expression; black squares represent median levels of gene expression; red squares represent higher than median levels of gene expression. Legend units: 1.0 = differs from median probe intensity by one log 2 unit (2-fold).
(TIF)

Table S1 miRNA gene profiles in cultured neural progenitor cells isolated from the SVZ in rats subject to 7 days of MCAo.
(XLS)

Table S2 Biologic functions associated with commonly upregulated and downregulated miRNAs in neural progenitor cells after MCAo.
(XLS)

Table S3 Primer sequences.
(XLS)
