# Supplementary figures and images for: MicroRNA Profiling in Subventricular Zone after Stroke: MiR-124a Regulates Proliferation of Neural Progenitor Cells through Notch Signaling Pathway
Source: PLoS One. 2011 Aug 26;6(8):e23461. doi: 10.1371/journal.pone.0023461 (PMC3162555; doi:10.1371/journal.pone.0023461)

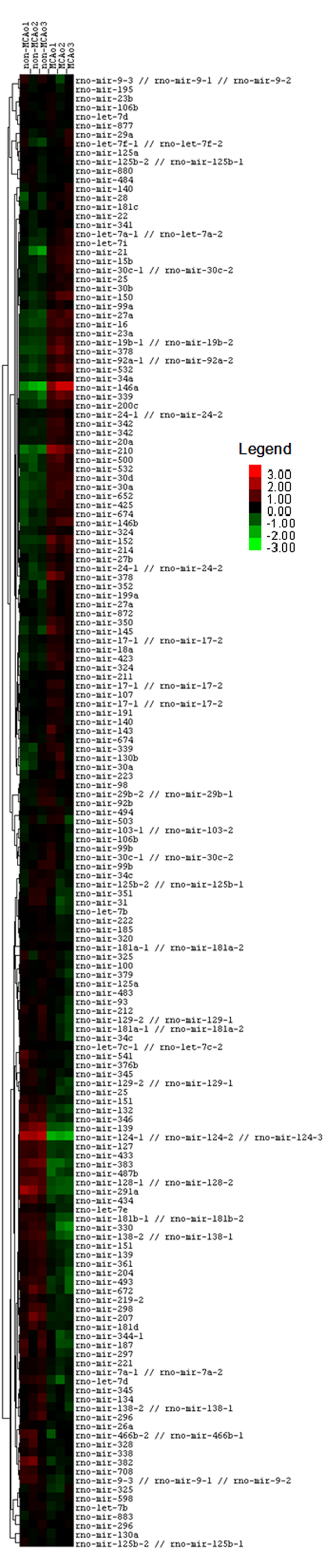

Supplement: Figure S1 — Cluster diagram for detected microRNA from Affymetrix microRNA microarray experiment. MiRNA probe expression values (Log2 transformed & normalized microarray probe intensities) of detected miRNA in either MCAo non-MCAo samples were median centered. Each column represents a single sample, and each row represents a single miRNA probe. Green squares represent lower than median levels of gene expression; black squares represent median levels of gene expression; red squares represent higher than median levels of gene expression. Legend units: 1.0 = differs from median probe intensity by one log 2 unit (2-fold). (TIF) [file pone.0023461.s001.tif]
